# How AI can be used to promote public and population health

**DOI:** 10.3389/fpubh.2026.1773572

**Published:** 2026-01-29

**Authors:** William B. Weeks, Juan M. Lavista Ferres

**Affiliations:** Microsoft AI for Good Lab, Redmond, WA, United States

**Keywords:** artificial intelligence, collaboration, population health, public health, research

## Abstract

Here, we summarize the work that Microsoft's philanthropic Artificial Intelligence (AI) for Good Lab has completed in the realm of promoting public and population health. In particular, after providing examples of how the AI for Good Lab has articulated the value of using AI to improve public and population health, we provide examples and references of the work demonstrating how the Lab has: applied Artificial Intelligence (AI) to improve maternal, fetal, and infant health; leveraged large language models to improve population health; and applied AI to improve rural health and healthcare. We also summarize what we have learned through our work, finding that: getting the question right and ensuring the limitations of any analysis are understood is important; collaboration across public, private, and educational institutions with subject matter experts will be the most effective and efficient way to harness this new technology; and that focusing on metrics that reflect health, and not just the accuracy of the model, is the most impactful way to improve the health of populations, worldwide.

## Introduction

Healthcare delivery and public health efforts seek to improve the health of the population served. While healthcare delivery is an important aspect of population health, public health is even more so: only about 20% of a population's health status can be attributed to health care delivery, with the remaining 80% attributed to genetics and public health measures that include health behaviors and social determinants of health, defined as the non-medical factors that affect health outcomes. While prior public health research has relied on traditional regression and other analytic methods, using artificial intelligence (AI) to address public health issues offers a relatively new approach that is able to explore non-linear and interactive relationships more adroitly.

Given the complexities of the interactions of healthcare delivery, genetics, health behaviors, and social determinants of health, AI may be able to uncover important insights regarding relationships between measures of public health and health outcomes. Should such relationships be revealed, policymakers wanting to improve population health might focus their efforts on addressing those health behaviors or social determinants that most impact health status. Such geographically- or specified-population-targeted efforts could not only use scarce resources more efficiently but also generate a measurable health-related return on that investment. For example, while research has demonstrated that improving local economic conditions can reduce local morbidity and mortality rates, AI can help model the timing, magnitude, and beneficiaries of those potential returns so that policymakers can make informed investment decisions about which interventions should be undertaken to generate the greatest returns to health over a specified period.

To reveal those relationships, since 2020, Microsoft's philanthropic AI for Good Lab has dedicated resources to exploring how AI can be used to address the biggest problems in sustainability, humanitarian action, and health (1). The Lab includes (generally PhD-level) Microsoft-employed data and computer scientists who collaborate with not-for-profit organizations (like universities, charitable organizations, and government agencies) to apply AI methods to research intent on solving those problems. As a segment of the broader literature examining how to use AI to promote public and population health, this paper shows how this Lab and these researchers have contributed to the peer-reviewed literature addressing health and healthcare challenges in the following five areas.

### Articulating the potential uses of AI in public and population health

AI for Health researchers have contributed to the literature by articulating the promise and perils of applying AI methods to improve population health. These contributions include editorials asserting how AI might be used to advance public health (2), why AI should be considered a public service (3), and how AI methods might be used to improve health and public health (4).

In addition, AI for Health researchers have emphasized the centrality and importance of population health in efforts to achieve sustainable development goals (5) while also cautioning that inaccurate storytelling used to persuade public health practitioners and policymakers about the importance and relevance of public health concerns may mislead decision makers (6).

AI for Health researchers have also explored how healthcare delivery might be improved using AI methods. For example, they have described the promise and peril of the use of AI in achieving the quadruple aim in healthcare [enhancing the patient and provider experience, improving and equitably distributing health care quality and outcomes, and reducing per-capita healthcare costs (7)] as well as how AI might help address the underperformance of the United States healthcare system (8).

### Using AI to improve maternal, fetal, and infant health

AI for Health researchers have made substantial contributions to the literature regarding sudden unexpected infant death (SUID). SUID is an umbrella term that includes sudden infant death syndrome (SIDS), accidental suffocation and strangulation in bed, and other deaths from unknown causes that occur in infants less than 1 year old; it is a devastating experience for a family.

To try to reduce the incidence of SUID, AI for Health researchers have used AI methods to explore the relationship between maternal smoking before and during pregnancy (9), the relationship between maternal infections in pregnancy (10), and the relationship between maternal obesity (11) and the risk of SUID. Further, AI for Health researchers have explored geographic variation in SUID within the United States (12) and identified distinct populations of SUID based on the age of the infant at death (13), including an analysis of SUID in the first week of life (14). Finally, AI for Health researchers found that rates of SUID changed during the COVID pandemic (15).

Beyond SUID, AI for Health researchers have identified risk factors for late gestational stillbirth in the United States (16), worked to improve the understanding of the relationship between fetal growth and late gestational stillbirth (17), and identified risk factors for postpartum hemorrhage and severe maternal morbidity in low-risk laboring populations (18).

Finally, AI for Health researchers have described how AI can be helpful: in addressing challenges in maternal, newborn, and child health in low resource settings so that policymakers can best use scarce resources (19); understanding how micro-geographic analysis can be used to identify locations where children experience high malnutrition risk in India so policymakers can target interventions to those who most need them (20); determining how AI—in concert with satellite data—can be used to anticipate surges in childhood malnutrition in Kenya so that policymakers can intervene to prevent it (21); and modeling the meningococcal antibody response following vaccination so that policymakers can determine when best to revaccinate (22).

### Using AI to explore relationships between social determinants of health and population health

As is well-known, the social determinants of health—the conditions in which people are born, grow, work, live, and age—have a greater impact on one's health than does the healthcare delivery system. Understanding that impact on individual and population health, AI for Health researchers have undertaken research to explore the specific relationships between social determinants of health and population health.

Having considered how social determinants of health might be used to address cardiovascular disease and health equity (23), AI for Health researchers have described the relationship between local economic distress and both life expectancy (24) and inequities in health outcomes, clinical care, health behaviors, and other social determinants of health (25). AI for Health researchers used this knowledge and machine learning tools to evaluate the relationships between social determinants of health and diabetes prevalence in New York City (26) and changes in local economic conditions and drug overdose deaths in the United States between 2000 and 2019 (27). The insights generated from examining these relationships support previous work suggesting that policymakers should consider using local economic stimulus to improve health and health outcomes (28).

Further, AI for Health researchers have explored the Medicare fee-for-service population, identifying discrepancies in coding practices that are meant to capture social determinants of health (29) and identifying where there are shortages of qualified proceduralists necessary to provide high-quality, best-outcomes procedural interventions (30).

AI for Health researchers have also used machine learning, social determinants of health data, and other data sources to forecast short-term drug overdose deaths in Connecticut (31), improve flu tracking (32), predict quality of life trajectories among patients with different types of cancer (33), and explore and challenge inherent assumptions when examining historical data when cultural norms have changed (34).

### Using large language models to improve population health

Large Language Models (LLMs) have the potential to dramatically impact healthcare delivery, public health interventions, and health outcomes. To advance this work, AI for Health researchers have demonstrated the efficacy of a conversational chatbot for cigarette smoking cessation (35), articulated how LLMs could be used to facilitate shared-decision making in the physician-patient encounter (36), demonstrated how LLMs can be used to quickly and efficiently create user-friendly applications for assessment of eligibility for Medicaid or other social services (37), to evaluate the accuracy of answers to nutritional questions in Brazilian Portuguese (38), to identify sources of misinformation in internet news stories (39), and to surface high-quality, evidence-based medical information in internet searches (40).

### Using AI to improve rural health and healthcare

To conclude a synopsis of the program's efforts examining health and public health, AI for Health researchers have been instrumental in providing a mapping tool and evaluating rural-urban disparities in health outcomes, clinical care, health behaviors, and social determinants of health (41). To address the health and economic disparities this work found, AI for Health researchers developed an action plan to leverage technology and AI to improve the health of those living in rural settings (42) and devised a hub-and-spoke model designed to improve rural health and healthcare access by facilitating clinical and technological partnerships between urban and rural hospitals (43).

## What we have learned

Our experiences have generated several insights that might be useful to others exploring the use of AI in public and population health. First, as in all research, it is critically important to get the question right. While “fishing” through data exploration might generate interesting hypotheses, it is critical to understand the analytic approach to be used, to appreciate the limitations of any datasets to be explored, and to obtain the correct permissions—including human subjects review, where appropriate—to conduct the research. Second, and a corollary to the first point, the questions that AI has the potential to answer are challenging and complex: it is therefore imperative to include subject matter experts (in public health and in the particular medical area being explored) in the development of the question, the research itself, the interpretation of findings, and the write-up. Third, to achieve the first two points requires collaboration with institutions that have the appropriate data for analysis and the subject matter expertise to conduct the research; selecting the best partners who are interested in sharing findings, regardless of what they are, in an effort to advance knowledge is paramount to success. Finally, research undertaken must have the potential to be impactful: researchers must apply their limited resources to questions that can improve public and population health; therefore, the metrics that should be evaluated should be reflective of health impact and not only measure of model accuracy.

## Conclusion

Here, we have shown how AI can be—and is being—used to promote public and population health. While AI for Health researchers have been incredibly productive in contributing to this area, we believe that this is just the beginning. In the future, we anticipate that AI will be used to integrate individualized healthcare delivery and social determinant of health intervention plans, estimate returns to investment in targeted social determinant of health interventions, use the Internet of Things to surveille for and intervene on infectious disease outbreaks, and use multi-modal data (such as satellite data) for epidemiological purposes, climate-change induced disease spread, and even concurrent evaluation of social determinant of health metrics.

We live in an exciting time. While AI is revolutionizing how many businesses operate, its greatest impact may be on improving public and population health. A comprehensive, strategic deployment of particular AI tools that target public and population health interventions, support administrators in montitoring those interventions, and inform policymakers on which interventions to choose can efficiently and effectively support a learning cycle, wherein individual and population health continuously improve.

## References

[1] Ferres JML Weeks WB. AI for Good: Applications in Sustainability, Humanitarian Action, and Health. Hoboken, NJ: John Wiley & Sons (2024).

[2] Weeks WB Taliesin B Lavista JM. Using artificial intelligence to advance public health. Int J Public Health. (2023) 68:1606716. doi: 10.3389/ijph.2023.1606716 38024205 PMC10651492

[3] Lavista Ferres JM Fishman EK Rowe SP Chu LC Lugo-Fagundo E. Artificial intelligence as a public service. J Am Coll Radiol. (2023) 20:919–21. doi: 10.1016/j.jacr.2023.01.013 37003310

[4] Ferres JL. AI Methods and their Application to Health and Prevention Using Open Data. Amsterdam Neuroscience - Cellular & Molecular Mechanisms, Integrative Neurophysiology. Amsterdam, NL: Vrije Universiteit Amsterdam (2023).

[5] Weeks WB Weinstein JN Lavista JM. All sustainable development goals support good health and well-being. Int J Public Health. (2023) 68:1606901. doi: 10.3389/ijph.2023.1606901 38205020 PMC10777740

[6] Weeks WB. Storytelling in scientific conferences: mitigating misinformation risk. Int J Public Health. (2024) 69:1607289. doi: 10.3389/ijph.2024.1607289 38689667 PMC11059058

[7] Weeks WB Lavista Ferres JM Weinstein JN. Artificial intelligence: promise and peril in achieving the quadruple aim in healthcare. Front Artif Intell. (2024) 7:1430756. doi: 10.3389/frai.2024.1430756 38962504 PMC11220197

[8] Weeks WB Rizk RC Rowe SP Fishman EK Chu LC. Unraveled: prescriptions to repair a broken health system. J Am Coll Radiol. (2024) 21:1919–21. doi: 10.1016/j.jacr.2024.01.021 38295920

[9] Anderson TM Lavista Ferres JM Ren SY Moon RY Goldstein RD Ramirez JM . Maternal smoking before and during pregnancy and the risk of sudden unexpected infant death. Pediatrics. (2019) 143:e20183325. doi: 10.1542/peds.2018-3325 30858347 PMC6564075

[10] Weatherly M Trivedi A Chembrolu R Gupta S Ramirez JM Lavista Ferres JM . Maternal infections in pregnancy and the risk of sudden unexpected infant death in the offspring in the U.S., 2011-2015. PLoS ONE. (2023) 18:e0284614. doi: 10.1371/journal.pone.0284614 37083949 PMC10121007

[11] Tanner D Ramirez JM Weeks WB Lavista Ferres JM Mitchell EA. Maternal obesity and risk of sudden unexpected infant death. JAMA Pediatr. (2024) 178:906–13. doi: 10.1001/jamapediatrics.2024.2455 39073792 PMC11287443

[12] Mitchell EA Yan X Ren SY Anderson TM Ramirez JM Lavista Ferres JM . Geographic variation in sudden unexpected infant death in the United States. J Pediatr. (2020) 220:49–55.e2. doi: 10.1016/j.jpeds.2020.01.006 32061407 PMC7995635

[13] Lavista Ferres JM Anderson TM Johnston R Ramirez JM Mitchell EA. Distinct populations of sudden unexpected infant death based on age. Pediatrics. (2020) 145:e20191637. doi: 10.1542/peds.2019-1637 31818863 PMC6939839

[14] Anderson TM Ferres JML Ramirez JM Mitchell EA. Sudden unexpected postnatal collapse resulting in newborn death in the United States. Am J Matern Child Nurs. (2021) 46:130–6. doi: 10.1097/NMC.0000000000000711 33587345 PMC8349372

[15] Tanner D Ramirez JM Weeks WB Ferres JML Mitchell EA. COVID-19 pandemic era changes in sudden unexpected infant death in the United States. Pediatr Open Sci. (2025) in press. doi: 10.1542/pedsos.2024-000364

[16] Tanner D Murthy S Lavista Ferres JM Ramirez JM Mitchell EA. Risk factors for late (28+ weeks' gestation) stillbirth in the United States, 2014-2015. PLoS ONE. (2023) 18:e0289405. doi: 10.1371/journal.pone.0289405 37647261 PMC10468071

[17] Tanner D Lavista Ferres JM Mitchell EA. Improved estimation of the relationship between fetal growth and late stillbirth in the United States, 2014-15. Sci Rep. (2024) 14:6002. doi: 10.1038/s41598-024-56572-7 38472269 PMC10933328

[18] Lengerich BJ Caruana R Painter I Weeks WB Sitcov K Souter V. Interpretable machine learning predicts postpartum hemorrhage with severe maternal morbidity in a lower-risk laboring obstetric population. Am J Obstet Gynecol MFM. (2024) 6:101391. doi: 10.1016/j.ajogmf.2024.101391 38851393

[19] Tadesse GA Ogallo W Cintas C Speakman S Walcott-Bryant A Wayua C. Bridging the gap: leveraging data science to equip domain experts with the tools to address challenges in maternal, newborn, and child health. npj Women's Health. (2024) 2:13. doi: 10.1038/s44294-024-00017-z

[20] Kim R Bijral AS Xu Y Zhang X Blossom JC Swaminathan A . Precision mapping child undernutrition for nearly 600,000 inhabited census villages in India. Proc Natl Acad Sci USA. (2021) 118:e2025865118. doi: 10.1073/pnas.2025865118 33903246 PMC8106321

[21] Tadesse GA Ferguson L Robinson C Kuria S Wanyonyi H Murage S . Forecasting acute childhood malnutrition in Kenya using machine learning and diverse sets of indicators. PLoS ONE. (2025) 20:e0322959. doi: 10.1371/journal.pone.0322959 40367047 PMC12077733

[22] Nasir M Weeks WB Gholami S Marfin A Alderson M Leader T . Modeling protective meningococcal antibody responses and factors influencing antibody persistence following vaccination with MenAfriVac using machine learning. PLoS ONE. (2025) 20:30323384. doi: 10.1371/journal.pone.0323384 40367245 PMC12077764

[23] McNeill E Lindenfeld Z Mostafa L Zein D Silver D Pagán J . Uses of social determinants of health data to address cardiovascular disease and health equity: a scoping review. J Am Heart Assoc. (2023) 12:e030571. doi: 10.1161/JAHA.123.030571 37929716 PMC10727404

[24] Weeks WB Chang JE Pagán JA Adamson E Weinstein J Ferres JML. The ecology of economic distress and life expectancy. Int J Public Health. (2024) 69:1607295. doi: 10.3389/ijph.2024.1607295 39132383 PMC11309997

[25] Weeks WB Chang JE Pagán JA Aerts A Weinstein JN Ferres JL. An observational, sequential analysis of the relationship between local economic distress and inequities in health outcomes, clinical care, health behaviors, and social determinants of health. Int J Equity Health. (2023) 22:181. doi: 10.1186/s12939-023-01984-6 37670348 PMC10478428

[26] Tanner D Zhang Y Chang JE Speyer P Adamson E Aerts A . Machine learning to evaluate the relationship between social determinants and diabetes prevalence in New York City. BMJ Public Health. (2024) 2:e001394. doi: 10.1136/bmjph-2024-001394 40018588 PMC11816513

[27] Saba SK Ferres JL Weeks WB. Leveraging public data: changes in local economic distress and drug overdose deaths at the county level, 2000-2019. Int J Public Health. (2025) 70:1607991. doi: 10.3389/ijph.2025.1607991 40060022 PMC11884982

[28] Weeks WB Lavista Ferres JM Weinstein JN. Health and wealth in America. Int J Public Health. (2024) 69:1607224. doi: 10.3389/ijph.2024.1607224 38559467 PMC10979796

[29] Chang JE Smith N Lindenfeld Z Weeks WB. Hospital use of common Z-codes for medicare fee-for-service beneficiaries, 2017-2021. Health Aff Sch. (2024) 2:qxad086. doi: 10.1093/haschl/qxad086 38756404 PMC10986262

[30] Weeks WB Demuro B Lee DM Michael D Weinstein JN Ferres JML. Choosing your surgeon wisely: using provider-procedure-volume-specific data for informed choice. Jn Community Med Public Health. (2024) 8:478. doi: 10.29011/2577-2228.100478

[31] Mukherjee S Becker N Weeks WB Ferres JL. Using internet search trends to forecast short term drug overdose deaths: a case study on Connecticut. In: 19th IEEE International Conference on Machine Learning and Applications. Miami, FL (2020). doi: 10.1109/ICMLA51294.2020.00208

[32] Wojcik S Bijral AS Johnston R Lavista Ferres JM King G Kennedy R . Survey data and human computation for improved flu tracking. Nat Commun. (2021) 12:194. doi: 10.1038/s41467-020-20206-z 33419989 PMC7794445

[33] Gauthier J Furtuna B Mangiavacchi J Gholami S Ferres JL Dodhia R . Novel data analytics identify predictors of quality-of-life trajectories in patients with AML or high-risk myeloid neoplasms. Blood. (2022) 140:5254–7. doi: 10.1182/blood-2022-165437

[34] Ferres JL Nasir M Bijral A Subramanian SV Weeks WB. Modeling to explore and challenge inherent assumptions when cultural norms have changed: a case study on left-handedness and life expectancy. Arch Public Health. (2023) 81:137. doi: 10.1186/s13690-023-01156-6 37495995 PMC10369838

[35] Bricker JB Sullivan BM Mull KE Lavista-Ferres J Santiago-Torres M. Efficacy of a conversational chatbot for cigarette smoking cessation: protocol of the QuitBot full-scale randomized controlled trial. Contemp Clin Trials. (2024) 147:107727. doi: 10.1016/j.cct.2024.107727 39490766 PMC11620904

[36] Elwyn G Ryan P Blumkin D Weeks WB. Meet generative AI… Your new shared decision-making assistant. BMJ Evid Based Med. (2024) 29:292–5. doi: 10.1136/bmjebm-2023-112651 38866469

[37] Ratna S Weeks WB Ferres JL Chopra A Pereira M. A Methodology for using large language models to create user-friendly applications for medicaid redetermination and other social services. Int J Public Health. (2024) 69:1607317. doi: 10.3389/ijph.2024.1607317 39220672 PMC11361957

[38] Abib SB Beracco RT Weeks WB. A Comparative study of the performance of copilot, Gemini, and ChatGPT answering diet and nutrition questions in Brazilian Portuguese and English. Microsoft J Appl Res. (2025) 22:13–21.

[39] Fein W Shapiro JN White K Ferres JML Weeks WB. Internet news stories on COVID-19 or vaccines published by reliable and unreliable sources. Microsoft J Appl Res. (2025) 22:296–304.

[40] Weeks WB Ghosh A Pant A Nayak P Ferres JML. An algorithm for surfacing high-quality evidence-based medical studies in internet searches. Microsoft J Appl Res. (2025) 22:344–50.

[41] Weeks WB Chang JE Pagán JA Lumpkin J Michael D Salcido S . Rural-urban disparities in health outcomes, clinical care, health behaviors, and social determinants of health and an action-oriented, dynamic tool for visualizing them. PLOS Glob Public Health. (2023) 3:e0002420. doi: 10.1371/journal.pgph.0002420 37788228 PMC10547156

[42] Weeks WB Spelhaug J Weinstein JN Ferres JML. Bridging the rural-urban divide: an implementation plan for leveraging technology and artificial intelligence to improve health and economic outcomes in Rural America. J Rural Health. (2024) 40:762–5. doi: 10.1111/jrh.12836 38520683

[43] Babu S Weinstein J Ferres J Weeks W. Improving rural healthcare by creating academic- and non-academic-rural hospital partnerships based on community health needs assessments and technological needs. J Rural Health. (2025) 41:e12927. doi: 10.1111/jrh.12927 39865945

